# Autoantibodies to Cytosolic 5′-Nucleotidase 1A in Primary Sjögren’s Syndrome and Systemic Lupus Erythematosus

**DOI:** 10.3389/fimmu.2018.01200

**Published:** 2018-06-05

**Authors:** Anke Rietveld, Luuk L. van den Hoogen, Nicola Bizzaro, Sofie L. M. Blokland, Cornelia Dähnrich, Jacques-Eric Gottenberg, Gunnar Houen, Nora Johannsen, Thomas Mandl, Alain Meyer, Christoffer T. Nielsen, Peter Olsson, Joel van Roon, Wolfgang Schlumberger, Baziel G. M. van Engelen, Christiaan G. J. Saris, Ger J. M. Pruijn

**Affiliations:** ^1^Department of Neurology, Center for Neuroscience, Donders Institute for Brain, Cognition and Behaviour, Radboud University Medical Center, Nijmegen, Netherlands; ^2^Laboratory of Translational Immunology, Department of Rheumatology and Clinical Immunology, University Medical Center Utrecht, Utrecht University, Utrecht, Netherlands; ^3^Laboratorio di Patologia Clinica, Ospedale San Antonio, Azienda Sanitaria Universitaria Integrata di Udine, Tolmezzo, Italy; ^4^Institute for Experimental Immunology, Euroimmun AG, Lübeck, Germany; ^5^Service de physiologie et d’explorations fonctionnelles, Service de rhumatologie, Centre de référence des maladies auto-immunes rares and Fédération de médecine translationnelle de Strasbourg, Université de Strasbourg, Strasbourg, France; ^6^Department of Autoimmunology and Biomarkers, Statens Serum Institut, Copenhagen, Denmark; ^7^Department of Clinical Sciences Malmö, Lund University, Malmö, Sweden and Department of Rheumatology, Skåne University Hospital, Malmö, Sweden; ^8^Copenhagen Lupus and Vasculitis Clinic, Centre for Rheumatology and Spine Disease, Rigshospitalet, Copenhagen University Hospital, Copenhagen, Denmark; ^9^Department of Biomolecular Chemistry, Radboud Institute for Molecular Life Sciences and Institute for Molecules and Materials, Radboud University, Nijmegen, Netherlands

**Keywords:** Cytosolic 5′-nucleotidase 1A, anti-cN-1A, NT5C1A, autoantibodies, inclusion body myositis, Sjögren’s syndrome, systemic lupus erythematosus

## Abstract

**Introduction:**

Autoantibodies to cytosolic 5′-nucleotidase 1A (cN-1A; NT5C1A) have a high specificity when differentiating sporadic inclusion body myositis from polymyositis and dermatomyositis. In primary Sjögren’s syndrome (pSS) and systemic lupus erythematosus (SLE) anti-cN-1A autoantibodies can be detected as well. However, various frequencies of anti-cN-1A reactivity have been reported in SLE and pSS, which may at least in part be explained by the different assays used. Here, we determined the occurrence of anti-cN-1A reactivity in a large number of patients with pSS and SLE using one standardized ELISA.

**Methods:**

Sera from pSS (*n* = 193) and SLE patients (*n* = 252) were collected in five European centers. Anti-cN-1A, anti-Ro52, anti-nucleosome, and anti-dsDNA reactivities were tested by ELISA (Euroimmun AG) in a single laboratory. Correlations of anti-cN-1A reactivity with demographic data and clinical data (duration of disease at the moment of serum sampling, autoimmune comorbidity and presence of muscular symptoms) were analyzed using SPSS software.

**Results:**

Anti-cN-1A autoantibodies were found on average in 12% of pSS patients, with varying frequencies among the different cohorts (range: 7–19%). In SLE patients, the anti-cN-1A positivity on average was 10% (range: 6–21%). No relationship was found between anti-cN-1A reactivity and the presence or absence of anti-Ro52, anti-nucleosome, and anti-dsDNA reactivity in both pSS and SLE. No relationship between anti-cN-1A reactivity and duration of disease at the moment of serum sampling and the duration of serum storage was observed. The frequency of muscular symptoms or viral infections did not differ between anti-cN-1A-positive and -negative patients. In both disease groups anti-cN-1A-positive patients suffered more often from other autoimmune diseases than the anti-cN-1A-negative patients (15 versus 5% (*p* = 0.05) in pSS and 50 versus 30% (*p* = 0.02) in SLE).

**Conclusion:**

Our results confirm the relatively frequent occurrence of anti-cN-1A in pSS and SLE patients and the variation in anti-cN-1A reactivity between independent groups of these patients. The explanation for this variation remains elusive. The correlation between anti-cN-1A reactivity and polyautoimmunity should be evaluated in future studies. We conclude that anti-cN-1A should be classified as a myositis-associated-, not as a myositis-specific-autoantibody based on its frequent presence in SLE and pSS.

## Introduction

Autoantibodies are often helpful in the diagnosis and follow up of patients with inflammatory myopathies. Traditionally these antibodies are characterized as myositis-specific (MSA) or myositis-associated antibodies (MAA), according to their specificity. In 2013, two independent research groups described a novel antibody in sporadic inclusion body myositis (IBM): anti-cytosolic 5′-nucleotidase 1A (anti-cN-1A; anti-NT5C1A) (1, 2). cN-1A is an enzyme involved in the conversion of adenosine monophosphate to adenosine, and it has a role in the dephosphorylation of nucleotides to nucleosides (1). IBM is a slowly progressive muscle disease with a late onset. Its cause is yet unknown; inflammation, degeneration, and mitochondrial dysfunction all seem to play a role in the pathogenesis of IBM. Anti-cN-1A is present in 33–76% of IBM patients, and the variation is probably not only due to differences between cohorts, but is also dependent on the detection method and cutoff values that are used (1). Anti-cN-1A testing can improve the diagnostic process in IBM, and it can be used as a marker of expected disease severity. Anti-cN-1A positive IBM patients have more pronounced bulbar weakness and a higher mortality rate (2, 3). The presence or absence of anti-cN-1A antibodies in IBM is not related to the duration of symptoms or to the presence or absence of other autoimmune diseases or other autoantibodies (2). The specificity of anti-cN-1A antibodies has been established in previous studies. In healthy controls and in patients with polymyositis, dermatomyositis, and other neurological diseases, the prevalence of anti-cN-1A is low (0–4%) (4). However, in the systemic autoimmune diseases primary Sjögren’s syndrome (pSS) and systemic lupus erythematosus (SLE) anti-cN-1A autoantibodies have been detected at various frequencies with different methods of detection (4–7). We aimed to establish the occurrence of anti-cN-1A reactivity in multiple independent groups of European pSS and SLE patients using a single standardized detection method.

## Materials and Methods

### Patients

Sera from pSS (*n* = 193) and SLE patients (*n* = 252) were collected in five different European centers: Tolmezzo, Italy; Strasbourg, France; Utrecht, The Netherlands; Malmö, Sweden and Copenhagen, Denmark. The patients were enrolled in biobanks in each of the participating centers, for which ethical permission was obtained. The SLE patients were diagnosed using the 1997 American College of Rheumatology criteria; pSS patients fulfilled the American-European Consensus Classification Criteria (8, 9). Demographic data (age and sex of the patient), clinical data (duration of disease at the moment of serum sampling, autoimmune co-morbidity, and presence of muscular symptoms) and the total duration of storage of the sample were retrieved from the respective biobank databases by the local researcher blinded for anti-cN-1A status. Muscular symptoms were defined as myalgia and muscle weakness, autoimmune comorbidity was defined as the presence of any other autoimmune disease. Patients with Sjögren’s syndrome secondary to SLE were classified as SLE.

### Laboratory Analysis

Anti-cN-1A, anti-dsDNA, anti-nucleosomes, and anti-Ro52 reactivities were tested by ELISA in a single laboratory. The anti-cN-1A, anti-dsDNA-NcX, and anti-nucleosomes ELISA are commercially available ELISAs and were performed according to the manufacturer’s instructions (respective order numbers EA 1675-4801G, EA 1572-9601G, and EA 1574-9601G, Euroimmun AG, Lübeck, Germany). The anti-cN-1A ELISA is based on recombinant full-length cN-1A antigen as described earlier (7). Results were evaluated semi-quantitatively as a ratio (optical density (OD) 450 sample/OD450 calibrator, ratio ≥ 1 positive). The anti-dsDNA-NcX ELISA utilizes native dsDNA (isolated from calf thymus) as antigen, which is immobilized via highly purified mononucleosomes free of histone H1, Scl-70, and other non-histone components (cutoff: ≥ 100 IU/ml) (10). The anti-nucleosomes ELISA is based on native mononucleosomes free of histone H1, Scl-70 and non-histone components (cutoff: ≥ 20 RU/ml) (11).

Determination of anti-Ro52 reactivity was performed using an in-house ELISA (Euroimmun). Microtiter plates (Nunc, Denmark) were coated with 1 µg/ml recombinant Ro52 in PBS, pH 7.5 overnight at 4°C, washed with PBS-0.05% (w/v) Tween-20, and blocked for 2 h with PBS-0.1% (w/v) casein, followed by washing. Sera diluted 1:200 in PBS-0.1% (w/v) casein were incubated for 30 min before washing. Bound antibodies were detected using anti-human IgG peroxidase conjugate (Euroimmun) and stained with tetramethylbenzidine (Euroimmun) for 15 min. OD was determined at 450 nm (reference 620 nm) using an automated spectrophotometer (Spectra Mini, Tecan, Germany). All procedures were carried out at room temperature. The cutoff of the anti-Ro52 ELISA was defined at the 99% percentile based on samples from healthy blood donors (*n* = 100), anti-nuclear antibodies-negative patients (*n* = 52) and rheumatoid arthritis patients (*n* = 40). Results were evaluated semi-quantitatively as a ratio (OD450 sample/OD450 calibrator, ratio ≥ 1 positive).

### Statistics

IBM SPSS for Windows version 22 (IBM Corp., Armonk, NY, USA) was used for statistical analyses. Chi-square tests and Fisher’s exact test (categorical variables) and Mann–Whitney *U* test (continuous, non-parametric variables) were used for pairwise comparisons between groups. Correlations between autoantibody titers and other variables (e.g., duration of storage) were analyzed using Spearman ranking. A 2-sided *p*-value of 0.05 or less was deemed statistically significant in this exploratory study.

## Results

**Figure 1:**
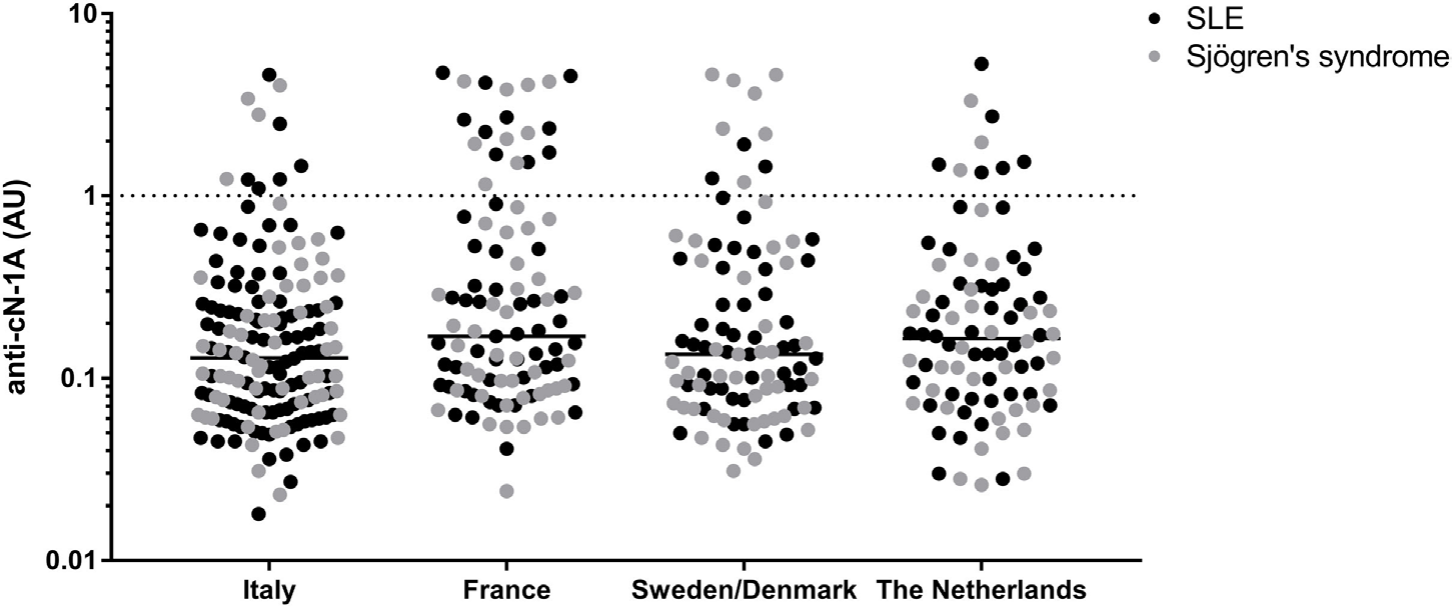
Distribution of anti-cN-1A reactivity in primary Sjögren’s syndrome and systemic lupus erythematosus (SLE) patients from different countries. Dotted line = cutoff of anti-cN-1A reactivity (1.0 AU).

Anti-cN-1A antibodies were found in 12% of the pSS patients (23/193) and in 10% of all SLE patients (26/252). The prevalence of anti-cN-1A showed some variation between countries in both diseases (Tables 1 and 2). The distribution of the levels of anti-cN-1A antibodies did not appear to differ between the groups from different countries and between pSS and SLE (Figure 1).

**Table 1:** Clinico-demographic correlations: anti-cN-1A in primary Sjögren’s syndrome (pSS).

pSS	Anti-cN-1A positive 12% (23/193)	Anti-cN-1A negative 88% (170/193)	*p*-Value
Provenance of the serum			0.21
– Italy	7%	93%	
– The Netherlands	8%	92%	
– France	19%	81%	
– Sweden	15%	85%	

Female/male	12%/18%	88%/82%	0.63

Presence of muscular complaints^b^	33% (4/12)	27% (20/74)	0.80

Presence of autoimmune co-morbidity^c^ (number of patients)	15% (3/20)	5% (7/135)	0.05^a^
– Antiphospholipid syndrome	– 4% (1)	– 0% (0)	
– Rheumatoid arthritis	– 4% (1)	– 2% (4)	
– Other	– 4% (1)	– 2% (3)	

Presence of current or past viral infection^d^	5% (1/19)	3% (4/128)	0.51

Presence of other antibodies			
– dsDNA	0% (0/23)	6% (11/170)	0.37
– anti-nucleosomes	0% (0/23)	6% (11/170)	0.37
– Ro52	65% (15/23)	68% (115/170)	0.82

*^a^Statistically significant (*p* ≤ 0.05)*.

*^b^Missing data in 55% of patients*.

*^c^Missing data in 20% of patients*.

*^d^Missing data in 24% of patients*.

**Table 2:** Clinico-demographic correlations: anti-cN-1A in systemic lupus erythematosus (SLE).

SLE	Anti-cN-1A positive 10% (26/252)	Anti-cN-1A negative 90% (226/252)	*p*-Value
Provenance of the serum			0.03^a^
– Italy	6%	94%	
– The Netherlands	12%	88%	
– France	21%	79%	
– Denmark	6%	94%	

Female/male	10%/17%	91%/83%	0.27

Presence of muscular complaints^b^	0% (0/19)	1% (2/160)	1.0

Presence of autoimmune co-morbidity^c^ (number of patients)	46% (11/24)	30% (58/195)	0.02^a^
– sSS	– 15% (4)	– 5% (10)	
– Antiphospholipid syndrome	– 19% (5)	– 19% (38)	
– Rheumatoid arthritis	– 12% (3)	– 2% (4)	
– Other	– 0% (0)	– 2% (4)	
– Combination	– 0% (0)	– 1% (2)	

Presence of current or past viral infection^d^	9% (2/23)	6% (10/177)	0.88

Presence of other antibodies			
– dsDNA	31% (8/26)	39% (88/226)	0.52
– anti-nucleosomes	23% (6/26)	31% (71/226)	0.50
– Ro52	42% (11/26)	32% (73/226)	0.38

*^a^Statistically significant (*p* ≤ 0.05)*.

*^b^Missing data in 29% of patients*.

*^c^Missing data in 13% of patients*.

*^d^Missing data in 21% of patients*.

The associations between anti-cN-1A reactivity and clinical, demographic, and laboratory findings are presented in Tables 1 and 2. A trend toward gender-association of anti-cN-1A reactivity did not reach statistical significance (18% of men in pSS and 17% in SLE showed anti-cN-1A reactivity, versus 12 (*p* = 0.5) and 10% (*p* = 0.2), respectively, of women). No association between anti-cN-1A and duration of disease or sample storage duration was found. Muscular complaints were almost equal for anti-cN-1A-positive and -negative patients, with myalgia being the most frequently reported symptom. One of the pSS patients had a biopsy-proven polymyositis, but this patient had no anti-cN-1A antibodies. In the anti-cN-1A-positive patients, a higher rate of autoimmune co-morbidity was seen: 15% of the anti-cN-1A-positive pSS and 50% of the anti-cN-1A-positive SLE patients suffered from one or more other autoimmune diseases, whereas autoimmune comorbidity was observed in 5 and 30%, respectively, of the anti-cN-1A-negative patients (*p* = 0.05 in pSS, *p* = 0.02 in SLE). The presence or absence of other antibodies did not differ between the anti-cN-1A-positive and -negative patients in both disease groups.

## Discussion

The current cohort with anti-cN-1A reactivity in 12% of pSS and 10% of SLE patients confirm the relatively high prevalence of anti-cN-1A in these diseases. In addition, a range of frequencies was observed in the groups from various European countries (pSS: 7–19%; SLE: 6–21%), which seems to be consistent with the variation in anti-cN-1A reactivity that was observed in these diseases in previous studies. However, it should be noted that the results of these studies were obtained with various in-house assays. Our study is the first to analyze anti-cN-1A in pSS and SLE patients from different centers in parallel using a single, standardized assay at a single laboratory. The current study does not offer an explanation for the relatively frequent presence of anti-cN-1A in pSS and SLE sera, nor for the variation in the frequency of anti-cN-1A among different countries.

Table 3 summarizes the results reported in four previous publications on anti-cN-1A reactivity in pSS and SLE. The largest cohort thus far consisted of 96 SLE and 44 pSS patients and the study included a comparison with clinical data as well. The subset of SLE patients with myositis (5%) did not show anti-cN-1A reactivity, and no correlation was found between anti-cN-1A reactivity and Raynaud’s phenomenon or interstitial lung disease. Similarly, among pSS patients no correlation could be found between anti-cN-1A status and clinical and laboratory features, and none of the pSS patients had any muscular complaints (6). Muro and coworkers reported concomitant positivity for anti-dsDNA and anti-Ro/SSA in the pSS and SLE patients with anti-cN-1A reactivity (5). The clinical and laboratory features of the two other reported cohorts are not described in detail (4, 7).

**Table 3:** Anti-cN-1A reactivity among systemic lupus erythematosus (SLE) and primary Sjögren’s syndrome (pSS) cohorts in former and current studies.

Cohort	Technique	Origin of samples	Disease	Number of patients	Anti-cN-1A positivity (%)
Herbert et al. (4)	ELISA with 3 synthetic peptides	The Netherlands	SLE	44	20
			pSS	22	36

Kramp et al. (7)	ELISA with recombinant full-length protein^a^	North American	SLE	33	6
			pSS	20	0

Lloyd et al. (6)	Immunoblotting against NT5C1A (full-length)-transfected and nontransfected HEK 293 cell lysates	USA	SLE	96	14
			pSS	44	23

Muro et al. (5)	ELISA with recombinant full-length protein	Japan	SLE	50	6
			pSS	50	4

Rietveld et al. (current study)	ELISA with recombinant full-length protein^a^	Europe	SLE	252	10
			pSS	193	12

*^a^The same standardized ELISA was used in these studies*.

Currently, IBM diagnosis is based on the combination of clinical features, laboratory findings, and muscle biopsy results (12). Unfortunately, application of the diagnostic criteria does not always lead to a quick and definite diagnosis. Although no treatment is yet available for IBM, a correct diagnosis is important, as for example misclassification as polymyositis and subsequent treatment with steroids can negatively influence the IBM disease course (13). The detection of anti-cN-1A antibodies could accelerate and improve the diagnosis of IBM. The presence of anti-cN-1A reactivity in a subset of SLE and pSS patients does not interfere with the clinical usefulness of anti-cN-1A testing in myositis due to the phenotypic differences between IBM and systemic autoimmune diseases. A standardized assay to detect anti-cN-1A antibodies, with clearly defined sensitivity and specificity, is of great importance before starting to use anti-cN-1A detection in clinical practice.

The large variation in the frequencies of anti-cN-1A in SLE and pSS reported in the aforementioned previous studies might be due to the different techniques that were used: western blotting and ELISA with the full-length recombinant protein produced in different host cells, and ELISA with three synthetic peptides [Table 3, reviewed in detail in Ref. (1, 14)]. The ELISA using three synthetic peptides is based on epitope mapping that has shown three regions of cN-1A that are targeted most frequently by autoantibodies. In that study, different patterns of reactivity with these three linear epitopes were observed (4). However, the use of small synthetic peptides does not allow the detection of antibodies against discontinuous or conformational epitopes. IBM sera reactive with one of these epitopes were not always positive when using full-length recombinant protein as antigen, whereas other sera were not reactive with any of the epitopes, but were positive when assessed using the full-length cN-1A ELISA (1). Variable seropositivity was seen in IBM patients dependent upon which isotype (IgG, IgA, or IgM) of anti-cN-1A antibody was tested (15, 16). In general, immunoblotting with full-length cN-1A expressed in transfected HEK293 cells showed a higher sensitivity and lower specificity than the three-peptide cN-1A ELISAs (1, 7). A direct comparison of the methodologies to detect anti-cN-1A antibodies has not yet been undertaken.

The role of cN-1A in the pathophysiology of IBM and the possible pathways of anti-cN-1A antibody induced pathology are not yet fully elucidated, although a recent study confirmed a role of anti-cN-1A antibodies in the onset of IBM (14, 17). *In vitro* and *in vivo* (in mice) passive immunization with anti-cN-1A–positive IgG leads to histological changes in the muscle fibers resembling the changes in IBM: an increase of p62 aggregates and an associated macrophage infiltration was seen in the *in vivo* model (17). Whether passive immunization led to pathophysiological changes as seen in SLE and pSS, is not stated. The variation in anti-cN-1A reactivity between the different countries included in our current study might be due to the different genetic backgrounds of the patients, although HLA-association studies in IBM did not show a difference between anti-cN-1A-positive and -negative patients (18).

The retrospective nature of our study led to some difficulties in the interpretation of the clinical data. First of all, for some items a large subset of data is lacking, for example regarding the presence or absence of muscular complaints. Furthermore, the presence or absence of muscular symptoms might be subject to reporting bias of patients: fatigue and diffuse pain in patients with systemic autoimmune diseases could be reported as myalgia. Autoimmune comorbidity might have been reported in different ways and antiphospholipid syndrome, for example, might not have been reported in a subset of patients. This means that the finding of an increased rate of autoimmune comorbidity in the anti-cN-1A-positive patients should be interpreted with caution. A prospective study with standardized clinical data collection and a broader panel of autoantibodies (including for example anti-CCP, anti-thyroid, and anti-skin autoantibodies) should clarify the relationship between anti-cN-1A reactivity and the presence of comorbidities, in particular other autoimmune diseases. A former study on IBM, using standardized data extraction sheets, did not show such a correlation (2). The included sera were provided by European centers only, meaning that comparisons with cohorts with other ethnical backgrounds can be difficult. We did not test healthy subjects in parallel with the SLE and pSS patients, but two independent laboratories have previously evaluated healthy subjects using the same ELISA as we have used in this study, showing anti-cN-1A reactivity in 2 and 3% (1/52 and 7/202) (7).

This retrospective study confirms the relatively high prevalence and substantial variation in anti-cN-1A reactivity in different cohorts of pSS and SLE patients. Based on this, we conclude that anti-cN-1A should be classified as a MAA, not as a MSA. Prospective studies should shed more light on the role of anti-cN-1A in pSS and SLE to elucidate its pathophysiological role and to further explore its potential correlation with clinical features.

## Ethics Statement

Local ethics committee approval concerning the pSS and SLE biobanks is present in each of the participating centers (Lund University 2012/98; UMC Utrecht METC 12-296; Strasbourg CCP Est IV 09-02-2010, Italy: Authorization of the Privacy Guarantor No. 9, December 12th, 2013).

## Author Contributions

Initiation and design of this research: AR, CS, BE, and GP. Clinical data collection, establishment of the patient groups, and contribution of cases: NB, AM, LH, SB, JR, JG, GH, CN, PO, and TM. Establishment of the antibody detection method and laboratory analysis: WS, NJ, CD, BE, and GP. Statistical analysis: AR and LH. Draft manuscript preparation: AR. All authors were involved with the review of the manuscript and approved the final version.

## Conflict of Interest Statement

GP and BE are inventors of a patent (EP20120740236) licensed to Euroimmun AG and GP received financial support from Euroimmun for his research programme. CD, NJ, and WS are employees of Euroimmun AG. WS is a board member of Euroimmun AG. WS and CD are shareholders of Euroimmun AG. The remaining authors declare that the research was conducted in the absence of any commercial or financial relationships that could be construed as a potential conflict of interest.
